# Cerebral Venous Thrombosis Due to Overdrainage in a Patient With Normal Pressure Hydrocephalus: A Case Report

**DOI:** 10.7759/cureus.28721

**Published:** 2022-09-03

**Authors:** Ana Rita Silva, Mariana Santos, Maria João Machado, Ricardo Moreira, José Nuno Alves, Célia Machado, Ana Filipa Santos, Carla Ferreira, Ricardo Maré

**Affiliations:** 1 Neurology Department, Hospital de Braga, Braga, PRT; 2 Neuroradiology Department, Hospital de Braga, Braga, PRT; 3 Neurosurgery Department, Hospital de Braga, Braga, PRT

**Keywords:** overdrainage, cerebrospinal fluid shunt, cerebrospinal fluid (csf), ventriculoperitoneal shunting, normal pressure hydrocephalus, cerebral venous thrombosis

## Abstract

Mechanical shunting of cerebrospinal fluid (CSF) is an effective treatment for hydrocephalus but is not exempt from complications. A 67-year-old male with a history of normal pressure hydrocephalus (NPH) and ventriculoperitoneal shunting (VPS) one year ago presented with gait disturbance and memory impairment. His head computed tomography (CT) was normal, and the shunting pressure was reduced from 110 to 70 mmH_2_0 with gait and memory improvement. One week later, he reported persistent pressure headaches, which worsen when lying down, accompanied by nausea and vomiting. His neurological examination was notable for a short-stepped wide-based gait. Two generalized seizures were observed. CT cerebral venography revealed sinus venous thrombosis (SVT). After two days, a new CT was performed, and bilateral subdural hygromas were found. The shunting pressure was readjusted to 110 mmH_2_0, and symptom improvement was noted. One week later, CT showed enlargement and bleeding of subdural collections. The drainage system was closed, and the patient continue to recover.

The temporal association between pressure adjustment and symptom onset and the evidence of progressive subdural effusions suggest that the decrease of CSF volume by overdrainage led to an increase in cerebral blood volume and the dilatation of the venous sinus, which precipitated thrombus formation.

## Introduction

Normal pressure hydrocephalus (NPH) is a reversible cause of subcortical dementia and parkinsonism. It is characterized by progressive gait dysfunction, cognitive impairment, and urinary incontinence, although the full triad is not necessary for diagnosis. Ventriculoperitoneal shunting (VPS) of cerebrospinal fluid (CSF) is the only effective treatment for hydrocephalus [1].

Mechanical shunting of cerebrospinal fluid is an effective treatment for hydrocephalus but is not exempt from complications [2], such as postural headache, subdural hematoma, shunt tube obstruction or migration, mechanical failure, meningitis, or abdominal complications. Here, we report a case of sinus venous thrombosis (SVT) due to overdrainage in the long-term follow‑up of an NPH shunt patient.

## Case presentation

A 66-year-old male complained of a one-year history of progressive gait disturbance that progressively led to the use of a cane. He noticed difficulty with turning and postural instability without freezing. He denied tremors, slowness of movement, swallowing difficulty, olfactory loss, urinary incontinence, or constipation. There was also no history of mental impairment, hallucinations, delusions, or dream enactment. His previous medical history was irrelevant. His family history was unremarkable. No new medication had been started, and no exposure to neuroleptics, carbon monoxide, or toxics was recorded. On examination, he scored 25/30 on Montreal Cognitive Assessment (lost one point on the clock‑drawing task, one point on the animal naming task, and three points on delayed recall). Speech and language were normal. He walked slowly with a wide base and took 25 steps and 19 seconds to complete 10 meters. He needed four steps to turn 180 degrees and took two steps back on the pull test. His proprioception was normal, and he had no cerebellar, parkinsonian, or pyramidal signs.

**Figure 1:**
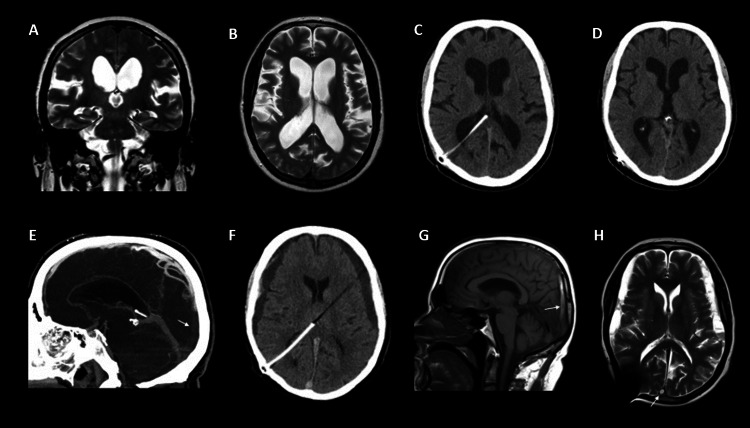
Cerebral venous thrombosis due to overdrainage in a patient with normal pressure hydrocephalus (A) Coronal and (B) axial T2-weighted (T2W) MRI shows ventriculomegaly (Evan’s index superior to 0.3 and acute callosal angle) and disproportionated changes in the subarachnoid spaces (dilated Sylvian fissures and narrow sulci at the high convexity). (C) The first control CT after ventriculoperitoneal shunting reveals correct placement. (D) Three weeks later, a new control CT shows a reduction of the ventricular system. (E) CT venography depicts a filling defect in the posterior superior sagittal sinus (white arrow). (F) Control CT shows bilateral frontal subdural effusions. (G) Midsagittal T1-weighted (T1W) MRI shows a high signal within the posterior superior sagittal sinus consistent with subacute thrombosis (white arrow). (H) Axial T2W MRI reveals slight enlargement and bleeding of subdural collections and a slight hyperintensity within the posterior superior sagittal sinus (white arrow), consistent with thrombosis and partial recanalization of the sigmoid sinus.

His brain magnetic resonance imaging (MRI) (Figure 1A, 1B) showed ventriculomegaly and disproportionate changes in subarachnoid spaces, suggesting NPH. His spinal cord MRI was normal. Electroencephalography (EEG) revealed bilateral frontal dysfunction without epileptiform activity. A high-volume lumbar puncture (40 mL) was performed, and improvement in his gait balance and turns was observed. The opening pressure was 18 mmH_2_0, and the CSF analysis was normal. Due to clinical improvement, a diagnosis of NPH was made, and he underwent VPS (Polaris®; opening pressure: 110 mmH_2_0) (Figure 1C). Postural headaches were present in the first month post‑surgery but then gradually improved without valve adjustments. Control computed tomography (CT) scan showed a reduction in ventricular size (Figure 1D), and normal gait was restored at six months.

At the 12-month follow-up visit, he presented with an abnormal gait, and his son complained of memory loss and disorientation for the last few weeks. Head CT revealed no significant changes, and the shunt was patent and functioning. The shunting pressure was adjusted from 110 to 70 mmH_2_0 with gait and memory improvement. One week later, he reported acute onset of a new drug-resistant persistent pressure headache, which worsened when lying down or getting up from the chair and was associated with nausea and vomiting. He had no fever. Neurological examination was notable for a short-stepped wide‑based gait. At the emergency department, two generalized tonic‑clonic seizures were observed. Non-contrast head CT showed spontaneous hyperdensity of the superior sagittal sinus, torcula, and right lateral sinus. CT venography (Figure 1E) confirmed extensive venous thrombosis. Low-molecular-weight heparin and levetiracetam were initiated. Two days later, CT was performed, and bilateral frontal subdural effusions were found (Figure 1F). The shunting pressure was readjusted to 110 mmH_2_0, and symptom improvement was noted. One week later, his brain MRI showed slight enlargement and acute bleeding of subdural collections (Figure 1G, 1H). The drainage system was closed, and the patient continued to recover. Other causes of SVT were excluded. The patient was discharged two weeks later with a modified Rankin score of 2. At 90 days, the patient was nearly back to normal with a modified Rankin score of 1.

## Discussion

NPH symptoms are caused by mechanical pressure or the distortion of ventricular enlargement in the frontal lobes and basal ganglia. VPS promotes the diversion of CSF to the peritoneal cavity and the reduction of ventricular volume. Shunting overdrainage is reported in 5%-20% of patients [2-4], mostly in the first month post-shunting. It is defined by CSF hypotension syndrome and subdural effusion or slit ventricle on CT/MRI. Increasing the opening pressure is usually sufficient to solve the problem; however, in some cases, the shunt has to be removed. To prevent excessive drainage, the initial opening pressure of the valve is intentionally set high and then slowly reduced in the first months of follow-up according to the clinical course. In the majority of cases, the correct valve setting is encountered within the first three months [2].

This was also the case in this patient, who was initially symptomatic in the first month post‑shunting and then fully adapted to normal function. Nevertheless, on the 12‑month follow-up, the previous symptoms from hydrocephaly returned. At this point, it was assumed that the system was underfunctioning, the reason why a reduction of the opening pressure was tried. Nevertheless, this reduction was excessive and perhaps too abrupt, originating an overdrainage syndrome and subsequently SVT. What is noteworthy in this patient is that in the first days after shunting pressure reduction, he did not complain of postural headaches as in the first weeks post‑shunting, and his motor symptoms improved; however, one week later, he presented with an acute hypertension intracranial syndrome due to SVT.

Previous reports have proposed a reasonable mechanism for SVT in the context of spontaneous intracranial hypotension [5]. According to the Monro-Kellie hypothesis (the sum of the volume of intracranial blood, CSF, and brain remains constant), the decrease of CSF is accompanied by an increase in cerebral blood volume. To accommodate more volume, the venous structures dilate and the blood flow velocity is reduced. The consequent blood stasis then precipitates thrombus formation. The temporal association between pressure adjustment and symptom onset, the evidence of progressive subdural effusions, and the exclusion of other causes suggest that the rapid and excessive decrease of CSF volume was responsible for SVT in this patient. Fully recovering after closing the system also favored this hypothesis.

## Conclusions

VPS is an effective treatment for NPH but is not exempt from serious complications, including SVT. Long-term follow-up watching for symptoms of both hydrocephalus and intracranial hypertension is needed, and careful and slow adjustments should be made whenever necessary. The onset of a new headache or a change in the usual pattern (from intracranial hypotension to hypertension syndrome) and/or the appearance of other neurological symptoms/signs after the placement of a shunt or pressure adjustment should prompt rapid neuroimaging (CT with angiography) to rule out complications, such as SVT and subdural hematoma.
